# Antibody responses to single‐dose SARS‐CoV‐2 vaccination in patients receiving immunomodulators for immune‐mediated inflammatory disease

**DOI:** 10.1111/bjd.20479

**Published:** 2021-09-01

**Authors:** A. Al‐Janabi, Z. Littlewood, C.E.M. Griffiths, H.J.A. Hunter, H. Chinoy, C. Moriarty, Z.Z.N. Yiu, R.B. Warren

**Affiliations:** Dermatology Centre Salford Royal NHS Foundation Trust Manchester NIHR Biomedical Research Centre The University of Manchester Manchester M6 8HD UK; Dermatology Centre Salford Royal NHS Foundation Trust Manchester NIHR Biomedical Research Centre The University of Manchester Manchester M6 8HD UK; Dermatology Centre Salford Royal NHS Foundation Trust Manchester NIHR Biomedical Research Centre The University of Manchester Manchester M6 8HD UK; Dermatology Centre Salford Royal NHS Foundation Trust Manchester NIHR Biomedical Research Centre The University of Manchester Manchester M6 8HD UK; National Institute for Health Research, Manchester Biomedical Research Centre Manchester University NHS Foundation Trust The University of Manchester Manchester UK; Department of Rheumatology Salford Royal NHS Foundation Trust Manchester Academic Health Science Centre Salford UK; Centre for Musculoskeletal Research Faculty of Biology, Medicine and Health The University of Manchester Manchester UK; Affinity Biomarker Labs, Translation & Innovation Hub Building Imperial College London White City Campus London W12 0BZ UK; Dermatology Centre Salford Royal NHS Foundation Trust Manchester NIHR Biomedical Research Centre The University of Manchester Manchester M6 8HD UK; Dermatology Centre Salford Royal NHS Foundation Trust Manchester NIHR Biomedical Research Centre The University of Manchester Manchester M6 8HD UK

Dear Editor, There are few data on whether immunomodulatory therapy attenuates humoral response to vaccines for severe acute respiratory syndrome coronavirus 2 (SARS‐CoV‐2). Some vaccines require two doses for maximum protection, with international variation in dosing schedules. A 3‐month interval between doses is being used in the UK. We evaluated antibody titres to SARS‐CoV‐2 following initial‐dose BNT162b2 (Pfizer/BioNTech) or AZD1222 (AstraZeneca) vaccines in adults with psoriasis and other immune‐mediated inflammatory diseases (IMIDs) receiving biologic and/or oral nonbiologic immunomodulators.

Participants were recruited from Salford Royal NHS Foundation Trust. Blood samples were collected 2–12 weeks after the first vaccine dose. Total antibodies against SARS‐CoV‐2 spike protein S1 receptor‐binding domain were quantified using the Roche Elecsys Anti‐SARS‐CoV‐2 S immunoassay (Roche Diagnostics Limited, Burgess Hill, UK) and anti‐S1 IgG was measured using the Siemens SARS‐CoV‐2 IgG immunoassay (sCOVG) (Siemens, Munich, Germany). The Elecsys Anti‐SARS‐CoV‐2 immunoassay, which detects antibodies against the nucleocapsid antigen absent from vaccines, identified those with prior infection. Two multivariable logistic regression models, excluding patients with prior SARS‐CoV‐2 infection (*n* = 22), were created with positive or negative Elecsys anti‐S1 and sCOVG assays as outcome variables, medication category as the exposure and a priori confounders (Table 1). The study was approved by the London‐Surrey Borders Research Ethics Committee and Health Research Authority.

**Table 1:** Multivariable logistic regression and characteristics of 120 recruited participants by humoral response to SARS‐CoV‐2 vaccination

	Elecsys SARS‐CoV‐2 S (Roche)		sCOVG (Siemens) IgG		
	Pos. (≥ 0·8 U mL^−1^)	Neg. (< 0·8 U mL^−1^)	Pos. (≥ 1 U mL^−1^)	Neg. (< 1 U mL^−1^)
Total	102 (85)	18 (15)	71 (59)	49 (41)
Drug class^a^				
Biologic	73 (90)	8 (10)	55 (68)	26 (32)
Oral immunomodulator	23 (74)	8 (26)	10 (32)	21 (68)
Biologic and oral immunomodulator	6 (75)	2 (25)	6 (75)	2 (25)
Sex				
Male	60 (85)	11 (15)	44 (62)	27 (38)
Female	42 (86)	7 (14)	27 (55)	22 (45)
Age (years)				
18–39	21 (100)	0 (0)	17 (81)	4 (19)
40–59	56 (89)	7 (11)	42 (67)	21 (33)
≥ 60	25 (69)	11 (31)	12 (33)	24 (67)
Vaccine				
BNT162b2	55 (92)	5 (8)	39 (65)	21 (35)
AZD1222	47 (78)	13 (21)	32 (53)	28 (47)
Time from vaccine (days)				
0–28	37 (74)	13 (26)	29 (58)	21 (42)
≥ 29	65 (93)	5 (7)	42 (60)	28 (40)
Prior SARS‐CoV‐2^b^	22 (100)	0 (0)	22 (100)	0 (0)
No prior SARS‐CoV‐2	80 (82)	18 (18)	49 (50)	49 (50)
Logistic regression models (*n* = 98),^c^ odds ratio (95% confidence interval)						
Drug class^a^			Elecsys SARS‐CoV‐2 S assay	sCOVG IgG assay	
Biologic			Reference	Reference	
Oral immunomodulator		Not adjusted	0·30 (0·10–0·91)	0·15 (0·05–0·44)	
		Adjusted	0·31 (0·08–1·17)	0·18 (0·06–0·59)	
Biologic and oral immunomodulator		Not adjusted	0·13 (0·02–1·08)	0·62 (0·08–4·67)	
		Adjusted	0·06 (0·01–0·80)	0·61 (0·07–5·32)	

The data are presented as the number (%) of patients testing positive or negative, unless stated otherwise. ^a^Biologics included abatacept (1), adalimumab (29), brodalumab (3), certolizumab (2), etanercept (2), guselkumab (6), ixekizumab (7), risankizumab (4), secukinumab (6), tildrakizumab (1) and ustekinumab (20). Oral immunomodulators included apremilast (2), ciclosporin (2), dimethyl fumarate (7), methotrexate (16), methotrexate and tofacitinib combined (1) and prednisolone (3). Combinations of treatment included the following: apremilast and guselkumab (1), azathioprine and infliximab (1), dimethyl fumarate and guselkumab (1), methotrexate and adalimumab (1), methotrexate and etanercept (1), methotrexate and rituximab (1), methotrexate and ustekinumab (2). ^b^Prior infection as diagnosed by Elecsys nucleocapsid assay. ^c^The models excluded those with prior infection (*n* = 22). Confounders or covariates included age (continuous), sex, vaccine type and number days from vaccine dose to antibody test (continuous).

In total 120 participants with IMIDs were recruited, including psoriasis (*n* = 107), psoriatic arthritis (*n* = 25), rheumatoid arthritis (*n* = 10), systemic lupus erythematosus (*n* = 1) and Crohn disease (*n* = 3); some patients had more than one condition. The median age was 53 years (interquartile range 33–73) and the ethnicities of the recruited participants included white (*n* = 111) and Asian (*n* = 9). The median time from vaccination to venepuncture was 34 days (interquartile range 23–46). Our data show that 15% of patients with IMIDs receiving immunomodulators failed to mount a detectable antibody response to a single dose of the BNT162b2 or AZD1222 vaccines; 41% had no detectable anti‐S1 IgG. Anti‐SARS‐CoV‐2 S appears more sensitive than the sCOVG assay. Nonbiologic immunomodulators, for example methotrexate, reduced the odds of a detectable antibody response compared with biologics: adjusted odds ratio (OR) 0·31 [95% confidence interval (CI) 0·08–1·17] and OR 0·18 (95% CI 0·06–0·59) for the Elecsys Anti‐SARS‐CoV‐2 S and sCOVG assays, respectively (Table 1). This contrasts with data from healthy populations, which show close to 100% seroconversion 14–35 days after the first vaccine dose, as measured by Roche Elecsys Anti‐SARS‐CoV‐2 S or other spike‐antigen‐specific assays detecting IgG and total immunoglobulin.^1^–^5^ No similar data for the sCOVG assay are available.

All of our participants with prior COVID‐19 had antibodies detected by both assays after a single vaccination, in line with previous observations.^1^, ^2^ Increasing age was also associated nonlinearly with reduced odds of a positive antibody response: OR 0·12 (95% CI 0·03–0·46) and OR 0·12 (95% CI 0·04–0·39) for the Elecsys Anti‐SARS‐CoV‐2 S and sCOVG assays, respectively, in those aged 60 years and over compared with those aged below 60 years, in a separate post hoc analysis. This is consistent with phase I trial data for the BNT162b2 vaccine,^4^ and may be due to immunosenescence.

There have been few other studies examining the humoral response to SARS‐CoV‐2 vaccines in patients receiving immunomodulators. In 436 immunosuppressed solid organ transplant recipients, 17% developed anti‐S1 antibodies by 14–21 days following a single dose of mRNA vaccine, although there was no control group.^6^ Geisen *et al*. evaluated antibody responses following the second dose of mRNA vaccines in 42 controls and 26 patients with IMIDs, including psoriasis (*n* = 4), psoriatic arthritis (*n* = 2) and rheumatoid arthritis (*n* = 8), and others receiving biologics, conventional disease‐modifying agents and/or prednisolone. They showed reduced anti‐S IgG titres and SARS‐CoV‐2 neutralization in patients receiving immunomodulators, but no difference between those receiving tumour necrosis factor inhibitors and nonbiologic immunomodulators. However, all 26 participants receiving immunomodulators had IgG antibodies above the assay cutoff.^7^

Our data show that not all patients on immunomodulators mount a detectable humoral response after a single dose of the BNT162b2 or AZD1222 vaccines and might therefore remain susceptible to COVID‐19. Strengths of our study are the utilization of two assays and controlling for a range of confounders. Limitations include the lack of a control IMID group not receiving an immunomodulator, and the modest sample size, resulting in wide CIs for some ORs and preventing subgroup analysis of drug types. Further controlled studies examining antibody titres following dosing of both vaccines are required, along with studies to define titres that correspond with protection.

## Acknowledgments

We would like to thank the DISCOVER Group for their contribution to the study design, including Professor John McLaughlin, Professor Ian Bruce, Professor Anne Barton, Professor Jimmy Limdi and Professor Kimme Hyrich. A.A. is a Medical Research Council Clinical Research Fellow. C.E.M.G. is a National Institute for Health Research (NIHR) Emeritus Senior Investigator. H.C. and R.B.W. are supported by the NIHR Manchester Biomedical Research Centre Funding Scheme. Z.Z.N.Y. is an NIHR‐funded clinical lecturer.

## Author Contribution

**Ali Al‐Janabi:** Conceptualization (equal); Data curation (supporting); Formal analysis (lead); Funding acquisition (supporting); Investigation (equal); Methodology (equal); Project administration (lead); Supervision (supporting); Writing‐original draft (lead); Writing‐review & editing (equal). **Zoe Littlewood:** Data curation (lead); Investigation (equal); Methodology (equal); Project administration (equal); Writing‐review & editing (supporting). **Christopher Ernest, Maitland Griffiths:** Conceptualization (equal); Supervision (supporting); Writing‐review & editing (equal). **Hamish John, Alexander Hunter:** Conceptualization (equal); Data curation (supporting); Supervision (equal); Writing‐review & editing (equal). **Hector Chinoy:** Conceptualization (equal); Data curation (supporting); Writing‐review & editing (equal). **Chris Moriarty:** Investigation (equal); Methodology (equal); Resources (lead); Writing‐review & editing (supporting). **Zenas Zee Ngai Yiu:** Formal analysis (equal); Methodology (supporting); Visualization (supporting); Writing‐review & editing (supporting). **Richard B Warren:** Conceptualization (lead); Data curation (supporting); Formal analysis (supporting); Funding acquisition (lead); Investigation (equal); Methodology (equal); Supervision (lead); Writing‐review & editing (equal).
